# Psychological Well-Being and Short Video Flow in Adolescents: The Moderated-Mediation via Loneliness and Sensation-Seeking

**DOI:** 10.3390/bs16101750

**Published:** 2026-09-25

**Authors:** Samet Makas, Mustafa Baloglu

**Affiliations:** 1 Deparment of Guidance and Psychological Counselling, Faculty of Education, Sakarya University, Sakarya 54050, Türkiye; 2 Department of Special & Gifted Education, College of Education, United Arab Emirates University, Al Ain P.O. Box 15551, United Arab Emirates; baloglu@hotmail.com

**Keywords:** psychological well-being, loneliness, sensation seeking, short video flow, adolescents

## Abstract

In the digital age, adolescents are increasingly using short video apps. Examining the roles of loneliness and sensation seeking is critical to understanding this behavior. This study aims to examine the relationship between psychological well-being and short video flow in adolescents and to reveal the mediating role of loneliness and moderating role of sensation seeking in this relationship. The cross-sectional convenience sample comprised 373 adolescents aged 12–15 years, with 60.9% girls and 39.1% boys (mean age = 13.2, SD = 0.74), recruited from two schools in Turkey. Participants completed the Short Video Flow Scale, Short Sensation Seeking Scale for Adolescents, Psychological Well-being Scale, and UCLA Loneliness Scale Short Form For Adolescents. The hypothesized mediation pathways were tested using the Hayes PROCESS macro (Models 4 and 14). Loneliness plays a fully mediating role between psychological well-being and short video flow. Sensation seeking plays a moderating role between loneliness and short video flow. The findings suggest that psychological well-being may be indirectly associated with short video flow through these two potential explanatory variables. In particular, higher psychological well-being may be linked to lower sensation seeking and lower loneliness, and these processes may be associated with short video flow in different ways.

## 1. Introduction

In recent years, short video platforms have gained a significant place in the daily digital lives of teenagers. Platforms such as TikTok, Instagram Reels, and YouTube Shorts, in particular, are widely used due to their short-form content, constantly evolving stream structures, and user-specific recommendation systems (Bucknell Bossen & Kottasz, 2020; Conte et al., 2025). These platforms are used not only for entertainment but also for acquiring information, passing the time, establishing social interaction, and sharing daily experiences (Bucknell Bossen & Kottasz, 2020). Therefore, short video platforms have become a research area that has attracted attention in recent years for understanding teenagers’ digital behavior. Use experience is defined as an individual’s intense focus on the activity they are doing, their disregard for time, and their desire to continue the activity (Qin et al., 2022). In the context of short videos, consecutive content viewing is associated with spending longer than planned on the platform and a persistent tendency to consume more content (Qin et al., 2023). In this respect, short video use refers not only to time spent on the platform but also to the qualitative nature of the experience, including immersion, interaction levels, and state of flow.

Adolescence is a developmental period characterized by accelerated identity development, increased importance of peer relationships, and a greater inclination towards new experiences. During this period, online environments can become a significant part of adolescents’ social lives (Odgers & Jensen, 2020). Particularly with the increase in time spent in digital environments, short video platforms have become more visible among adolescents (Conte et al., 2025). The brevity of content, rapid changes, and presentation tailored to personal interests appear to align with the appeal of short video platforms among adolescents (Qin et al., 2022). These features can also facilitate the streaming experience, characterized by concentration, immersion in the content, and altered perception of time during viewing. Indeed, research on adolescents shows that the streaming experience in short video usage is a significant psychological process in terms of user behavior (Qin et al., 2023). However, it is noted that digital media experiences can differ among adolescents, and the same usage pattern may be associated with different psychological patterns (Beyens et al., 2024). Examining the intense interaction adolescents have with short video content in conjunction with their psychological well-being and social experiences can contribute to a better understanding of the psychological processes underlying this interaction. Furthermore, determining the role of individual characteristics in these processes is important for understanding the differences among adolescents.

### 1.1. The Relationship Between Psychological Well-Being and Short Video Flow

With the proliferation of short video platforms, the relationship between experiences on these platforms and individuals’ psychological characteristics has become an important area of research. Studies on digital media use show that evaluating online experiences solely based on usage duration is insufficient; the manner of use and the experience itself must also be considered (Dienlin & Johannes, 2020; Odgers & Jensen, 2020; Schønning et al., 2020). In this context, psychological well-being is considered a multidimensional construct encompassing an individual’s positive evaluations of their life, positive emotional experiences, and the ability to lead a functional life (Iasiello et al., 2020). The literature offers different explanations regarding the direction of the relationship between psychological well-being and digital media experiences. While it is widely accepted that digital media use may be related to psychological well-being, a reverse causality perspective points out that psychological difficulties may also be one of the antecedents of online orientation (Hartanto et al., 2021). From this perspective, it is suggested that individuals experiencing psychological difficulties may turn to online environments more in search of social connection, validation, or relief (Hartanto et al., 2021). Therefore, psychological well-being can be considered not only as a result affected by digital experiences, but also as a psychological characteristic that may be associated with these experiences.

In addition to the quantity of use on short video platforms, the psychological experience during use is also important. Flow Theory offers a significant conceptual framework for explaining this experience. Flow is defined as a state in which the individual is intensely focused on the activity they are performing, becomes engrossed in the experience, and may experience a change in their perception of time (Csikszentmihalyi, 1990; Nakamura & Csikszentmihalyi, 2002). Studies on short video platforms also address the flow experience through dimensions such as pleasure, concentration, and changes in time perception (Qin et al., 2022, 2023). Research, particularly on adolescents, shows that streaming is a significant component of the short video experience (Qin et al., 2023). Accordingly, short video streaming, as opposed to general or problematic short video use, refers to the intense engagement and immersion experienced during viewing. Therefore, it is thought that psychological well-being may be related to short video streaming. However, it is important to examine which psychological processes can explain this relationship.

### 1.2. The Mediating Role of Loneliness

Psychological well-being in adolescents is closely related not only to individual emotional states but also to social experiences. Specifically, low life satisfaction and positive affect can be associated with higher levels of loneliness and lower social belonging (Ryff, 1989; Iasiello et al., 2020). Loneliness is defined as a subjective and distressing experience stemming from a perceived incongruity between an individual’s current and desired social relationships (Dombak & Uğur, 2023; Maes et al., 2020; McKenna-Plumley et al., 2023). The increasing importance of peer relationships, social acceptance, and the need for belonging during adolescence makes loneliness particularly significant for this developmental stage (Chen et al., 2021; S. Liu et al., 2024; Verity et al., 2024). Indeed, research shows that loneliness in adolescents is associated with lower psychological well-being and various negative psychological outcomes (T. Liu et al., 2020; Loades et al., 2020). However, findings suggesting a reciprocal relationship between psychological well-being and loneliness indicate that the connection between these two constructs should not be considered a one-way process. Longitudinal findings indicate that the relationship between loneliness and psychological well-being in adolescents can be dynamic over time, and that the temporal ordering of these constructs should not be evaluated in a one-way manner (Verity et al., 2024). Therefore, loneliness may be associated with decreased psychological well-being, and conversely, low psychological well-being may predispose adolescents to experiences loneliness by reducing their satisfaction with social relationships and their sense of belonging.

One approach used to explain the relationship between loneliness and digital behaviors is the social compensation approach. According to this approach, individuals may turn to online communication to compensate for social needs that are not adequately met in offline environments (Valkenburg & Peter, 2007). In line with this explanation, it is suggested that psychological and social difficulties may not only be a consequence of digital media use, but also a precursor to online orientation (Hartanto et al., 2021). In the context of short video platforms, research on adolescents shows that the streaming experience is a significant component of interaction with the platform, and that dimensions of streaming such as pleasure, concentration, and changes in time perception are associated with problematic TikTok use (Qin et al., 2023). However, research directly examining the relationship between loneliness and short video streaming in adolescent samples is limited. When these findings and the social compensation approach are considered together, loneliness can be evaluated as a possible interpersonal mechanism that may explain the relationship between psychological well-being and short video streaming. Accordingly, it is predicted that the experience of loneliness associated with low psychological well-being may be linked to a tendency towards online environments, and that this tendency may be related to the streaming experience in the context of short videos. In this study, it is hypothesized that loneliness may play a mediating role in the relationship between psychological well-being and short video streaming.

### 1.3. The Moderating Role of Sensation-Seeking in the Relationship Between Loneliness and Short Video Flow

It is conceivable that the relationship between loneliness and short video streaming may not manifest at the same level in all adolescents. The Differential Susceptibility to Media Effects Model (DSMM) offers a theoretical framework for explaining this difference. The model suggests that media effects may differ depending on individuals’ dispositional, developmental, and social characteristics, and considers emotion-seeking as one of the dispositional susceptibility characteristics towards media use (Valkenburg & Peter, 2013). Emotion-seeking is a disposition characterized by a preference for novel, varied, intense, and stimulating experiences (Steinberg et al., 2008).

There are empirical findings supporting the relationship between emotion-seeking and digital behaviors. Research on adolescents shows that emotion-seeking is associated with problematic smartphone use (Pérez de Albéniz-Garrote et al., 2021). More directly in the context of short videos, Baltacı and Açar (2025) reported that emotion-seeking is associated with short video addiction in adolescents aged 14–19. While these findings do not directly address short video streaming, they suggest that emotional seeking is an individual difference that should be considered in adolescents’ interactions with short video platforms.

When these empirical findings are considered together with the DSMM’s assumption of individual sensitivity, it can be predicted that emotional seeking may differentiate the strength of the relationship between loneliness and short video streaming. While the social compensation approach explains why loneliness may be related to online engagement, DSMM suggests that media experiences associated with this engagement may differ according to individual characteristics (Valkenburg & Peter, 2007, 2013). Accordingly, it is hypothesized that the relationship between loneliness and short video streaming may be stronger in adolescents who have a higher propensity for novel and intense experiences.

### 1.4. Present Study

The increasing popularity of short video platforms among adolescents in recent years has necessitated a closer examination of the psychological processes associated with these platforms (Conte et al., 2025). Current studies show relationships between short video use and psychological well-being, loneliness, and online behaviors (Dienlin & Johannes, 2020; Qin et al., 2022; Zhao & Kou, 2024). However, the indirect paths connecting psychological well-being and short video flow, and how these associations may vary according to individual characteristics, have been examined in a limited number of studies (Conte et al., 2025). This study aims to examine the relationship between psychological well-being and short video flow use in adolescents. In this context, the mediating role of loneliness in the relationship between psychological well-being and short video flow, and the moderating role of sensation-seeking in the relationship between loneliness and problematic short video use, are evaluated. This model is consistent with current approaches arguing that digital behaviors are best understood not solely through direct links, but alongside broader psychological processes and individual differences (Dienlin & Johannes, 2020).

This study is significant in several respects. First, although short video platforms are becoming increasingly visible in adolescents’ daily lives, the psychological factors associated with these platforms have been examined in only a limited number of studies (Conte et al., 2025). In particular, the limited findings on the indirect patterns connecting psychological well-being and short video flow necessitate a more detailed examination of this relationship. Second, while digital behaviors are mostly examined through direct associations in the current literature, the role of indirect processes and individual differences has been relatively underexplored (Dienlin & Johannes, 2020). This study focuses on the relational path connecting psychological well-being and short video flow by examining the mediating role of loneliness. Finally, the observation that individuals reporting similar levels of loneliness do not use with short video platforms with uniform intensity highlights the relevance of individual differences. Overall, the study aims to offer a comprehensive perspective on short video usage by examining it alongside interrelated psychological and individual variables. Accordingly, the following hypotheses were tested:

**H1.**  *There is a negative relationship between psychological well-being and short video flow in adolescents.*

**H2.**  *Loneliness mediates the relationship between psychological well-being and short video flow.*

**H3.**  *Sensation seeking moderates the relationship between loneliness and short video flow.*

The hypothetical model is shown in Figure 1.

## 2. Materials and Methods

### 2.1. Participants and Procedure

The study included 373 middle school students aged 12–15 (M = 13.2, SD = 0.74). 227 of the participants were girls, and 146 were boys. By grade level, 206 students (55.2%) were in 7th grade, while 167 students (44.8%) were in 8th grade. Participants were selected using convenience sampling from two middle schools located in an urban area of Turkey. The inclusion criteria for the study were being between the ages of 12 and 15, being enrolled in 7th or 8th grade, using at least one platform that offers short-form video content, and voluntarily participating in the study. Students who did not use platforms offering short-form video content were excluded from the study.

A total of 400 students were initially invited to participate in the study. Of these students, 373 met the inclusion criteria and provided usable data, resulting in a response rate of 93.3%. A total of 27 students were excluded from the final analyses because they did not meet the inclusion criteria or because they completed the survey form incompletely or incorrectly. Since there were no missing data in the final dataset, no missing data handling methods were applied. Although participants’ use of short-form video platforms was one of the inclusion criteria for the study, no separate measurements were taken regarding daily usage time or frequency.

Data were collected between May and June 2025 after ethical approval was obtained from the “Sakarya University Ethics Committee for Educational Research and Publications” and the relevant school administrations. Prior to data collection, the purpose of the study was explained to the participants; it was stated that participation was voluntary, that no incentives would be offered, and that participant information would be kept confidential. Informed consent was obtained from all subjects involved in the study. The research forms were administered by the researcher in the classroom setting.

### 2.2. Measures

#### 2.2.1. Short Video Flow Scale

The scale, developed by Ye et al. (2023) to measure short video flow among adolescents, was adapted into Turkish by Türk and Yıldırım (2024). The scale consists of 8 items and uses a 5-point Likert scale ranging from “1 = Strongly Disagree” to “5 = Strongly Agree”. A higher total score indicates that participants derive more pleasure from short videos and spend more time watching them. The internal consistency coefficient, reported as 0.87 in the original study, was also 0.87 in Türk and Yıldırım (2024), who developed the Turkish form of the scale. This value proves that the Turkish form provides a reliable measurement. The Cronbach’s alpha (α) coefficient for the internal consistency of the scale in the current research sample was calculated as 0.84, while the McDonald’s omega (ω) coefficient was calculated as 0.84.

#### 2.2.2. Brief Sensation Seeking Scale (BSSS-4)

The scale, originally developed by Stephenson et al. (2003), was adapted into Turkish by Çelik (2015). The scale, which consists of 4 items and a single dimension, has a 4-point Likert scale ranging from “1 = Strongly Disagree” to “4 = Strongly Agree”. While the internal consistency coefficient was 0.66 (slightly below the conventional 0.70 threshold), in the original study, it was found to be 0.81 in the Turkish adaptation study. A high score from the scale means that adolescents have high sensation seeking. Importantly, in the current sample, the internal consistency coefficient was calculated as α = 0.83, confirming strong reliability for its use as a moderating construct despite its brief format. The Cronbach’s alpha (α) coefficient for the internal consistency of the scale in the current research sample was calculated as 0.83, while the McDonald’s omega (ω) coefficient was calculated as 0.83.

#### 2.2.3. Short Form of the UCLA Loneliness Scale (UCLS-SF)

The scale developed by Hays and DiMatteo (1987) to measure loneliness from adolescence to adulthood was adapted for Turkish culture by Yildiz and Duy (2014). Originally consisting of 8 items and a single dimension, the scale uses a 4-point Likert scale, with higher scores representing a more severe state of loneliness. In the adaptation study to Turkish culture, based on the findings of Exploratory Factor Analysis (EFA), one item with an insufficient factor loading was removed from the scale, resulting in its final 7-item version. Reliability studies of the measurement instrument showed acceptable reliability, with a Cronbach’s α of 0.74 and a test–retest correlation of 0.84. The Cronbach’s alpha (α) coefficient for the internal consistency of the scale in the current research sample was calculated as 0.78, while the McDonald’s omega (ω) coefficient was calculated as 0.85.

#### 2.2.4. Psychological Well-Being Scale

The Psychological Well-being Scale, developed by Diener et al. (2010) and adapted into Turkish by Telef (2013), is a unidimensional scale with 8 items on an 7-point Likert scale ranging from “1 = Strongly Disagree” to “7 = Strongly Agree”. The lowest possible score is 1, and the highest is 56. A higher score indicates a higher level of psychological well-being. The reliability score of the scale is stated as Cronbach’s α 0.80 (Telef, 2013). In the current sample of adolescents, the scale’s internal consistency coefficients were found to be α = 0.80 for Cronbach’s alpha and ω = 0.80 for McDonald’s omega. Furthermore, the results of confirmatory factor analysis indicated that the scale’s factor structure fit the current sample well (χ^2^/df = 2.10, CFI = 0.97, TLI = 0.96, RMSEA = 0.05, GFI = 0.96).

### 2.3. Data Analysis

For the analysis of the research data, the SPSS 31.0 package and the Hayes PROCESS macro (v4.2) were used. After calculating descriptive statistics between the variables, Pearson correlation coefficients were calculated to examine the relationships between the variables. Finally, Model 4 was used to determine the mediating role of loneliness in the relationship between psychological well-being and short video flow. Furthermore, Model 14 was used to test the moderating role of sensation seeking in the relationship between loneliness and short video flow. To evaluate the significance of mediating effects, 95% confidence intervals were constructed using the bootstrap method with 5000 resamples. A sensitivity power analysis was conducted using G*Power version 3.1.9.7 to assess the statistical adequacy of the sample size. Based on three predictor variables, a significance level of α = 0.05, and a statistical power of 0.80, it was determined that a sample of 373 participants would be sufficient to detect small effects at approximately f^2^ = 0.03.

## 3. Results

### 3.1. Preliminary Analyses

To evaluate the normality assumption, skewness and kurtosis coefficients were examined (Table 1). The observed skewness (−0.73 to 0.54) and kurtosis (−0.91 to 0.44) remain well within the ±1.5 limit, confirming that the data are normally distributed. Furthermore, no findings suggesting multicollinearity issues were observed among the variables. To check for multicollinearity, VIF, Tolerance, and Condition Index values were inspected. As shown, VIF values varied between 1.09 and 1.28, Tolerance ranged from 0.78 to 0.92, and Condition Index fluctuated between 1 and 17.59. The fact that these values meet the criteria of VIF < 10, condition index < 30, and tolerance > 0.10 indicates that multicollinearity does not pose a problem in the analyses (Tabachnick & Fidell, 2015).

When the correlation results in Table 1 are examined, it is seen that the short video flow is negatively related to psychological well-being (*r* = −0.27, *p* < 0.001), and positively related to loneliness (*r* = 0.49, *p* < 0.001) and sensation seeking (*r* = 0.37, *p* < 0.001). Psychological well-being was found to be negatively related to loneliness (r = −0.42, *p* < 0.001) and sensation seeking (*r* = −0.19, *p* < 0.001). A positive relationship was found between loneliness and sensation seeking (*r* = 0.28, *p* < 0.001).

### 3.2. The Mediating Role of the Loneliness

Hayes’ PROCESS Macro Model 4 analysis was applied to examine the mediating role of loneliness in the relationship between psychological well-being and short video flow. The results of the analysis are presented in Table 2.

According to the Model 4 and Figure 2 analysis, psychological well-being significantly and negatively predicts loneliness (β = −0.42, t = −8.96, *p* < 0.001). Whilst the direct effect of psychological well-being on short video flow was not found to be significant (β = −0.08, t = −1.63, *p* > 0.05), it was determined that loneliness significantly and positively predicted short video flow (β = 0.46, t = 9.14, *p* < 0.001). The results of the bootstrap analysis indicated that the indirect effect of psychological well-being on short video flow was significant (β = −0.19, 95% Boot CI [−0.25, −0.13]). This finding demonstrates that loneliness acts as a full mediator in the relationship between psychological well-being and short video flow.

### 3.3. Testing for the Moderated Mediation Effect of Sensation Seeking

Table 3 displays the results of Hayes’ Model 14 conditional process analysis, which explored whether loneliness mediates the relationship between psychological well-being and short video flow, and if this mediation varies by sensation seeking. The analysis shows that when loneliness, sensation seeking, and their interaction are considered together, they explain a significant portion of the variance in short video flow (R^2^ = 0.31, F = 42.02).

According to the Model 14 analysis, the effect of psychological well-being on short video flow (B = −0.04, *p* > 0.05), as well as the effects of loneliness (B = −0.05, *p* > 0.05), and sensation seeking (B = −0.21, *p* > 0.05) were not found to be significant. In contrast, the interaction effect between loneliness and sensation seeking was significant (B = 0.05, 95% CI [0.01, 0.08], t = 2.74). This finding indicates that the effect of loneliness on short video flow varies with the level of sensation seeking. Furthermore, the conditional effect of loneliness on short video flow was significant at low (−1 SD; B = 0.32, 95% CI [0.12, 0.53]), mean (B = 0.49, 95% CI [0.35, 0.63]), and high (+1 SD; B = 0.66, 95% CI [0.50, 0.81]) levels of sensation seeking. These results indicate that the positive association between loneliness and short video flow becomes stronger as sensation seeking increases. As shown in Figure 3, the relationship between loneliness and short video flow tends to increase significantly with rising sensation-seeking levels.

When the results of the conditional indirect effects were analysed, the indirect effect of psychological well-being on short video flow through loneliness was significant at low (−1 SD; B = −0.08, 95% Boot CI [−0.13, −0.02]), mean (B = −0.12, 95% Boot CI [−0.17, −0.08]), and high (+1 SD; B = −0.16, 95% Boot CI [−0.21, −0.11]) levels of sensation seeking. The bootstrap confidence intervals did not include zero at any of the three levels. The index of moderated mediation was also significant (Index = −0.012, Boot SE = 0.004, 95% Boot CI [−0.021, −0.004]) indicates that the magnitude of the indirect effect varies with levels of sensation seeking. The fact that the confidence interval does not include zero supports the view that the mediating role of loneliness in the relationship between psychological well-being and short video flow varies with levels of sensation seeking.

## 4. Discussion

The first hypothesis, H1 “There is a negative relationship between psychological well-being and short video flow in adolescents”, was supported. Accordingly, lower levels of psychological well-being were associated with higher reported short video flow. This finding aligns with prior literature indicating that lower subjective well-being and higher emotional distress are linked with greater digital immersion, such as negative correlations between flow-like engagement and happiness (Yayla & Çelikci, 2025). From a theoretical standpoint, adolescents experiencing lower psychological well-being may be more inclined to seek the immediate absorption and cognitive shift provided by short-video flow as a compensatory coping strategy. While the cross-sectional nature of this study precludes causal inferences regarding whether flow engagement further impacts daily routines or well-being over time, the observed statistical association highlights that lower baseline psychological well-being represents a key correlate of heightened digital flow immersion in adolescence.

The second hypothesis, H2 “Loneliness mediates the relationship between psychological well-being and short video flow “, was also supported. The findings indicate that loneliness serves as a mediating factor in the indirect association between lower psychological well-being and increased short video flow. A review of the literature reveals that short video use is generally associated with lower levels of loneliness but is also associated with reduced face-to-face interaction and weakened social participation (Zhang, 2026). Furthermore, while short video use is associated with loneliness, problematic short video use is negatively correlated with subjective well-being, as indicated in a meta-analysis of 58 studies (Tang et al., 2026). High engagement in digital environments may coincide with decreased face-to-face social involvement, which frequently overlaps with reports of loneliness. Thus, loneliness, alongside elevated digital use, forms a relevant relational context linked to broader psychological well-being.

The final hypothesis, H3 “Sensation seeking moderates the relationship between loneliness and short video flow”, was confirmed. Sensation seeking demonstrated a conditional role, such that the positive association between loneliness and short video flow was more pronounced among adolescents reporting higher sensation-seeking tendencies.

While limited literature directly addresses this three-way configuration, theoretical frameworks suggest that adolescents high in sensation seeking may display a stronger affinity for high-pace sensory input. Literature indicates that short video environments provide continuous novelty and stimulation (Su et al., 2021), which may closely align with the preferences of high sensation seekers, corresponding to longer durations of use. Upon disengaging from such dynamic digital stimuli, the lower pace of everyday offline environments may correspond to elevated reports of boredom or dissatisfaction, which tracks alongside higher subjective loneliness.

Conversely, adolescents lower in sensation seeking may exhibit diminished stimulation requirements, corresponding to lower engagement with short video flow. This pattern may reflect a higher degree of alignment with offline daily activities and less reliance on rapid digital content. In line with the Differential Susceptibility to Media Effects Model (Valkenburg & Peter, 2013), which posits that media-related outcomes vary based on individual differential susceptibility traits, these findings suggest that adolescents high in sensation seeking may exhibit greater conditional vulnerability to the rapid content flows of short video platforms when experiencing loneliness.

Research findings indicate a negative correlation between psychological well-being levels and short video flow among adolescents; therefore, education on responsible social media use, especially for adolescents, should be increased. Furthermore, promoting artistic and sporting activities that improve adolescents’ psychological well-being can help reduce deep psychological absorption in short video platforms. These activities, facilitated through school clubs or social clubs, can reduce feelings of loneliness and increase social support. Based on the research findings, rather than encouraging adolescents to spend long hours in digital environments, methods for coping with loneliness can be provided.

Additionally, mental health professionals working with adolescents in schools can prioritize adolescents with high sensation-seeking tendencies as a high-risk group when addressing short video flow or loneliness. Finally, adolescents with high sensation-seeking tendencies need to be encouraged to channel this craving into healthy activities. Some examples include adrenaline-pumping and exciting sports, esports teams, and outdoor activities.

The study has several limitations. First, the research is cross-sectional in design. This limits the generalizability of the research results. Secondly, the study is limited to a convenience sampling strategy applied to selected schools in a single urban area in Türkiye; this means that the findings cannot be generalized to adolescents with different cultural or socioeconomic backgrounds.

Furthermore, the variables used in the study were measured using data obtained from self-report scales. This is a limitation because adolescents may tend to rate socially positive items highly and negative items low. The study did not include objective or behavioral measurement tracking, and no specific information was collected about the types of platforms on which short video content was viewed. Finally, the study assessed the levels of short video flow among adolescents using items from the Short Video Flow Scale. In future research, detailed data on which platforms these short videos are used can be obtained.

Based on the research results, some suggestions for future research will be offered. First, since the current research is cross-sectional, there is a limitation to generalizing the results; therefore, future research could be designed longitudinally with the current variables. Additionally, experimental research could be conducted to determine the types of effects of short video flow on adolescents. These studies could compare levels of psychological well-being and loneliness between adolescents who use short videos and those who do not, or experimentally measure these levels in adolescents who use short videos and then stop using them. Furthermore, variables such as sleep quality, physical health, social support, and FOMO could be tested as mediators to understand better the statistical pathway linking adolescent short video flow and psychological well-being. Future research could also compare these variables in adolescents from different cultures.

## 5. Conclusions

This study investigated the mediating role of loneliness and the moderating role of sensation-seeking in the relationship between psychological well-being and short video flow in adolescents. High levels of short-form video use were associated with low levels of psychological well-being. Additionally, loneliness was shown to mediate the relationship between these two variables. However, this pattern was not uniform across adolescents; personality traits such as sensation-seeking played a moderating role in increasing this correlation. Adolescents with high sensation-seeking tendencies were found to be more vulnerable to the negative relationship between short video flow and loneliness. In conclusion, to improve adolescent well-being and reduce loneliness levels, it is suggested that attention should be focused not only on reducing short video flow but also on adolescents at high risk of sensation-seeking.

## Figures and Tables

**Figure 1:**
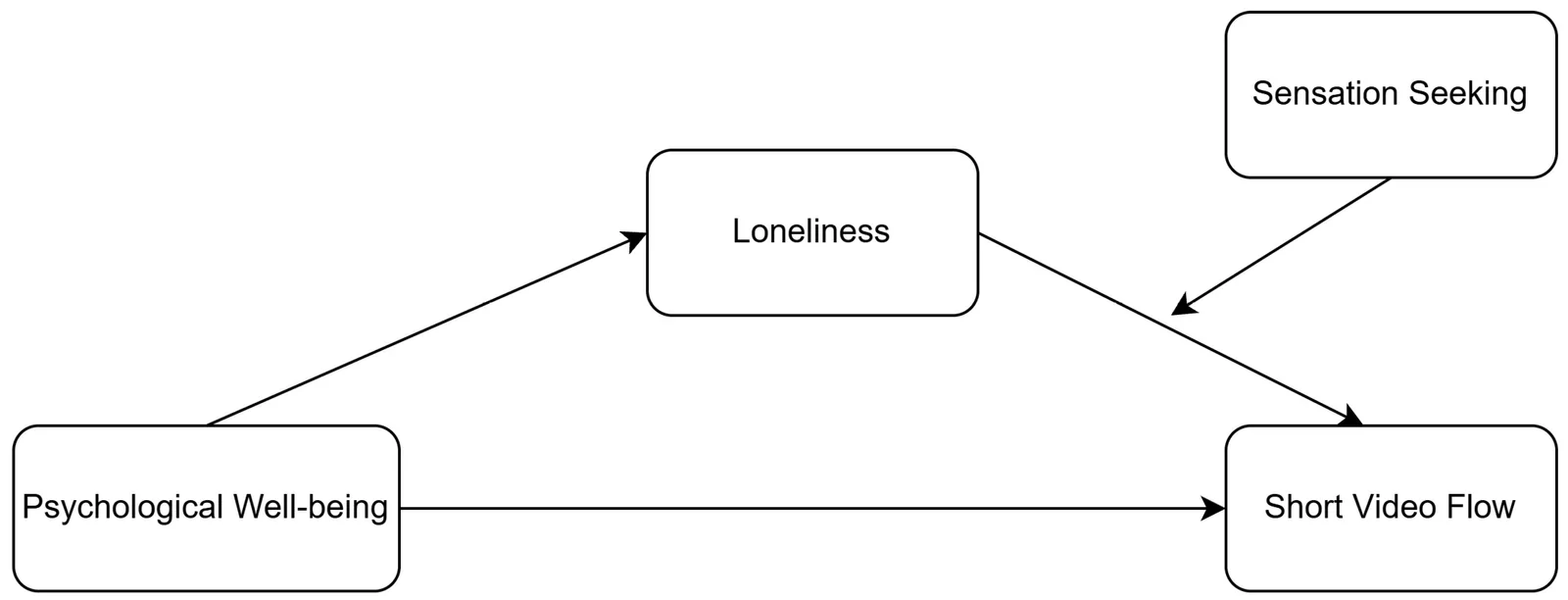
Hypothetical Model.

**Figure 2:**
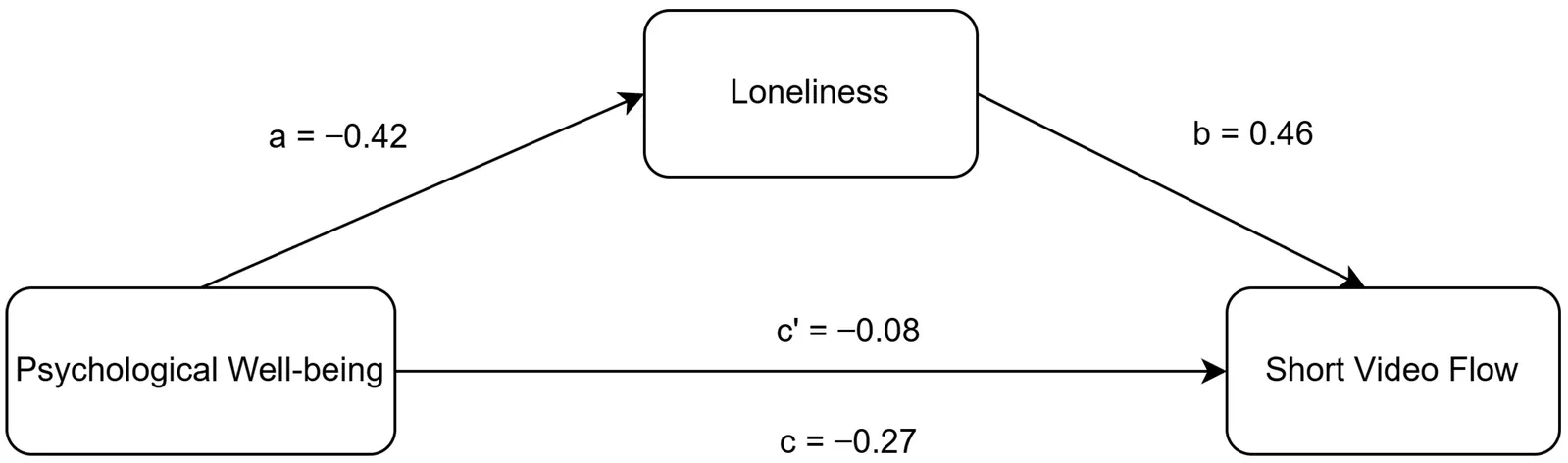
The Mediating Role of Loneliness in the Relationship Between Psychological Well-being and Short Video Flow.

**Figure 3:**
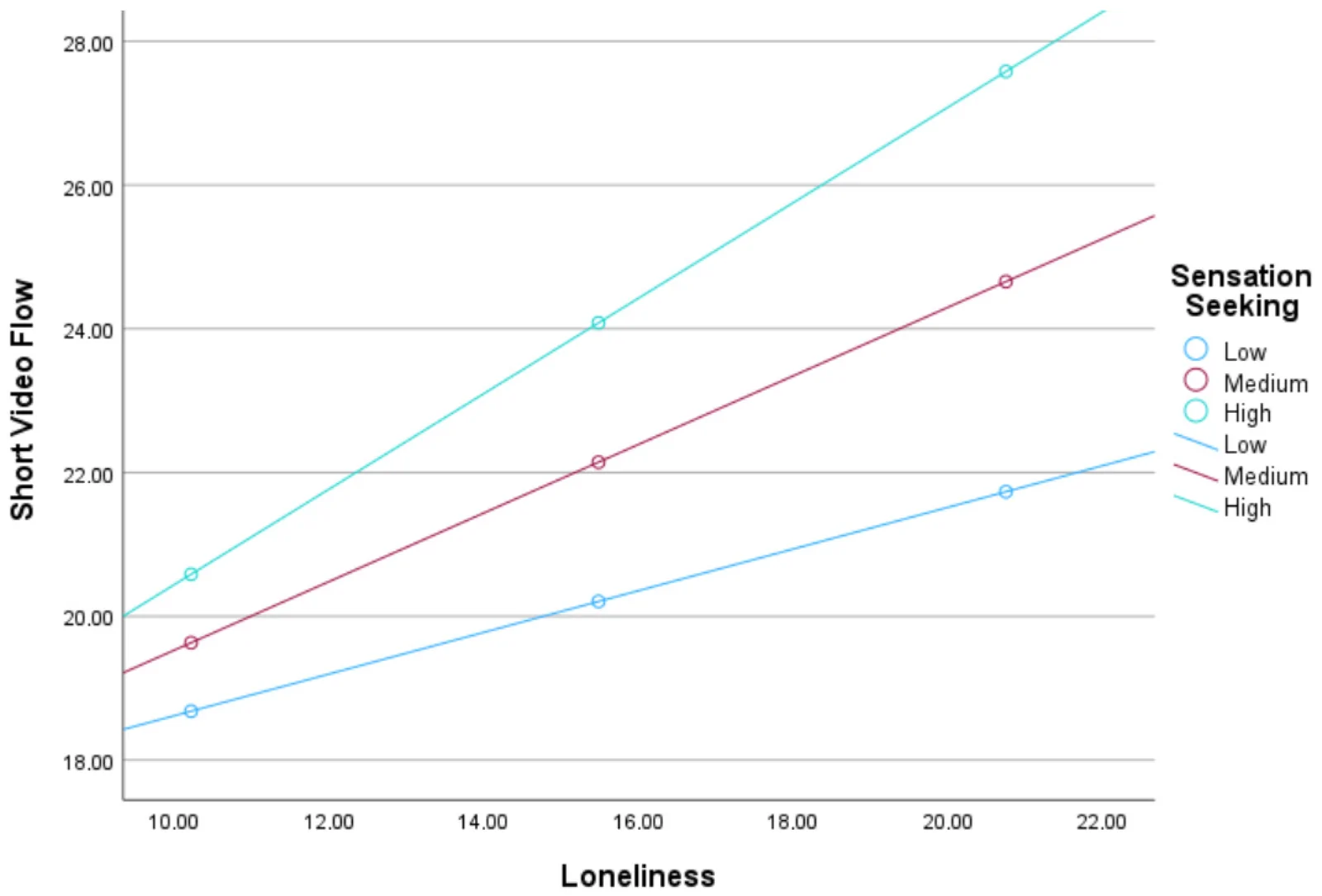
The moderating role of sensation seeking in the relationship between loneliness and short video flow.

**Table 1:** Results of the Correlation Analysis and Descriptive Statistics.

Variable	a	b	c	d	Mean	SD	Skew.	Kurt.	VIF	CI	Tol.
a: Short Video Flow	1				22.43	7.21	0.35	−0.57	-	1	-
b: Psychological Well-being	−0.27 **	1			39.06	9.06	−0.73	0.44	1.23	5.78	0.82
c: Loneliness	0.49 **	−0.42 **	1		15.48	5.27	0.54	−0.54	1.28	7.74	0.78
d: Sensation Seeking	0.37 **	−0.19 **	0.28 **	1	11.19	3.44	−0.31	−0.91	1.09	17.59	0.92

N: 373. ** *p* < 0.01.

**Table 2:** The mediating role of loneliness in the relationship between psychological well-being and problematic short video use.

Model	Loneliness (M)				Short Video Flow (Y)			
	B	β	SE	t	B	β	SE	T
Psychological Well-being (X)	−0.25	−0.42	0.027	−8.96	−0.06	−0.08	0.040	−1.63
Loneliness	-	-	-	-	0.62	0.46	0.063	9.14
Intercept/Constant	25.06	-	1.097	22.84	15.30	-	2.24	6.84
Model Summary	R^2^ = 0.18, F_(1,371)_ = 80.33, *p* < 0.001				R^2^ = 0.25, F_(2,370)_ = 60.10, *p* < 0.001			
Bootstrap Results for Indirect Effects				B	β	Boot SE	BootLLCI	BootULCI
Indirect Effect on Loneliness				−0.15	−0.19	0.031	−0.25	−0.13

Note. B = unstandardized coefficient; β = standardized coefficient.

**Table 3:** Results of the conditional mediation analysis of the loneliness in the relationship between psychological well-being and short video flow (Model 14).

Outcome	Predictors		R^2^	F	B	LLCI	ULCI	t
Short Video Flow	Intercept (Constant)				18.48	10.91	26.05	4.80 ***
	Psychological Well-being				−0.04	−0.11	0.04	−1.06
	Loneliness				−0.05	−0.50	0.39	−0.24
	Sensation Seeking				−0.21	−0.75	0.34	−0.74
	Loneliness × Sensation Seeking				0.05	0.01	0.08	2.74 **
	Model Summary		0.31	42.02				
Conditional Effects of Loneliness on Short Video Flow at Values of Sensation Seeking								
		Effect (B)	SE		LLCI		ULCI	
Low (−1 SD; 7.750)		0.32	0.11		0.12		0.53	
Mean (11.185)		0.49	0.07		0.35		0.63	
High (+1 SD; 14.620)		0.66	0.08		0.50		0.81	
Conditional Indirect Effects of Psychological Well-Being on Short Video Flow via Loneliness at Values of Sensation Seeking								
		Effect (B)	Boot SE		Boot LLCI		Boot ULCI	
Low (−1 SD; 7.750)		−0.08	0.03		−0.13		−0.02	
Mean (11.185)		−0.12	0.02		−0.17		−0.08	
High (+1 SD; 14.620)		−0.16	0.03		−0.21		−0.11	
Index of Moderated Mediation		Index	Boot SE		Boot LLCI		Boot ULCI	
Sensation Seeking		−0.012	0.004		−0.021		−0.004	

Note. ** *p* < 0.01; *** *p* < 0.001.

## Data Availability

The data that support the findings of this study are available from the corresponding author, upon reasonable request.
